# Increased Serum Signal Peptide-Complement C1r/C1s, Uegf, Bmp1 (CUB)-Epithelial Growth Factor Domain-Containing Protein-1 May Have a Role in the Pathophysiology of Late-Onset Fetal Growth Restriction

**DOI:** 10.3390/diagnostics15222891

**Published:** 2025-11-14

**Authors:** Gulseren Dinc, Suleyman Caner Karahan, Suleyman Guven

**Affiliations:** 1 Department of Obstetrics and Gynecology, Faculty of Medicine, Karadeniz Technical University, 61080 Trabzon, Turkey; sguven@ktu.edu.tr; 2 Medical Biochemistry, Faculty of Medicine, Karadeniz Technical University, 61080 Trabzon, Turkey; suleymancaner.karahan@ktu.edu.tr

**Keywords:** intrauterine growth restriction, ischemia-modified albumin, SCUBE-1

## Abstract

**Background/Objective**: Fetal growth restriction (FGR) is a condition in which the fetus fails to grow as expected for gestational age, and its growth is restricted. This study aimed to investigate the role of SCUBE-1, a new ischemia marker, in the diagnosis of late-onset FGR and its contribution to its pathophysiology. **Methods**: The data of 33 cases with late-onset fetal growth restriction were compared with the data of 33 cases without any pregnancy complications. Only cases beyond 32nd weeks of gestation were included. Fetal sonography and maternal/fetal vascular Doppler parameters were recorded. Maternal serum SCUBE-1 and ischemia-modified albumin (IMA) levels were measured. **Results**: The mean maternal was 29.82 ± 5.66 years old in the study group and 30.15 ± 4.85 years old in the control group. The gestational ages in both groups were comparable. Maternal serum IMA levels were found to be similar in the late-onset FGR and control groups. However, serum SCUBE-1 levels were found to be statistically significantly higher in the study group than in the control group. In cases where the SCUBE-1 level was above 1.64, the sensitivity for predicting late-onset FGR was 93.9% and the specificity was 60.6% (AUC 0.891, *p* < 0.001, 95% CI 0.811–0.970). **Conclusions**: This study reported a significant increase in SCUBE-1, which may be important in determining oxidative stress in late-onset FGR cases. SCUBE-1 was an effective and highly sensitive marker that could provide results in a maternal blood sample within an average of one hour and may be useful in the biochemical diagnosis of late-onset FGR. It may also be a useful serum marker for timing and planning delivery.

## 1. Introduction

Fetal growth restriction (FGR) is a condition in which the fetus fails to grow as expected for gestational age, and its growth is restricted. It occurs when the estimated birth weight (EFW), calculated based on ultrasonographic fetal measurements, is less than that expected for gestational age. According to guidelines published by internationally recognized authorities, this condition is characterized by a sonographic fetal abdominal circumference (AC) or EFW below the 10th percentile for gestational age [1,2]. A recent study reported that when the 32nd week of gestation was included in the definition and classification of FGR, the perinatal mortality, perinatal adverse pregnancy outcomes, and fetal neurological outcomes were different [3]. This study classified FGR diagnosed before 32 weeks as early-onset FGR and cases diagnosed after 32 weeks as late-onset FGR [4]. Perinatal hypoxia and related processes were reported to be less common in late-onset FGR [5].

FGR is a significant issue in maternal-fetal medicine because it increases the risk of perinatal mortality and morbidity. It can lead to intrauterine fetal death or stillbirth, and clinicians must be well aware of the potential for an early diagnosis through screening, which can prevent fetal death through timely delivery. In a study following 9150 pregnant women from the first trimester onward, a prevalence of 5% was observed. The late-onset form was seen in 5–10% of pregnancies [6].

Late-onset FGR cases present significant differences in etiology, pathophysiology, and fetal prognosis compared to early-onset cases. The underlying etiological factors and pathophysiological mechanisms of the late-onset type are not clearly known. The likelihood of placental pathology in these cases was reported to be relatively low, umbilical artery Doppler abnormalities were below 10% percent, and preeclampsia may present in approximately 15% of cases. However, angiogenic imbalance and hypoxia have been reported as partial contributors to the pathophysiology. It has been hypothesized that the underlying pathophysiological mechanism in these cases may be related to fetal central cardiovascular adaptation [5]. The reported maternal factors and fetal outcomes in early-onset vs. late-onset fetal growth restriction with odds ratios are summarized in Table 1 [7]. Accordingly, it can be argued that fetal cardiovascular and metabolic processes, as well as oxidative stress, may play a role in the pathophysiology of late-onset FGR. Therefore, further research is needed on this topic. The exact cause and pathophysiological mechanism of late-onset FGR remain unknown. The most commonly blamed hypothetical mechanism in this context is oxidative stress-mediated. Our research may contribute to the literature in this regard.

Treatment for these cases involves monitoring fetal growth with fetal biometry, fetal well-being with fetal biophysical scoring, non-stress testing, and fetal vascular Doppler studies. Treatment includes steroid administration to support fetal lung development and magnesium administration to support fetal neurological development and protection. Fetal well-being parameters, fetal Doppler, gestational age, and estimated fetal birth weight are evaluated, and delivery is planned at the appropriate time [1].

Numerous studies have examined the role of fetal cord oxidative stress markers in antenatal diagnosis of FGR. These included malondialdehyde, superoxide dismutase, catalase, ischemia-related metabolites (ischemia-modified albumin (IMA)), and hematological indices (homocysteine, nitric oxide, nucleated red blood cells). A recent meta-analysis on the subject concluded that many biomarkers are helpful in this context [8,9]. Specifically, Malondialdehyde, nucleated red blood cells, and ischemia-modified albumin were elevated, while superoxide dismutase and catalase levels were low in FGR cases, regardless of gestational age. However, no difference was found in homocysteine and nitric oxide levels in FGR cases compared to normal pregnancies [9]. In a recent review evaluating oxidative stress parameters in cord blood, total oxidant/antioxidant status, catalase, glutathione, ischemia-modified albumin, and nucleated red blood cells were found to be associated with fetal growth restriction. In contrast, reactive oxygen species/reactive nitrogen species, antioxidant enzymes, non-enzymatic antioxidants, and products of oxidative stress were not found to be significant in this context [8]. However, these analyses lacked a separate evaluation of early- and late-onset FGR cases and lacked data on the new ischemia marker SCUBE-1 (signal peptide-CUB (complement C1r/C1s, Uegf, and Bmp1)-epidermal growth factor-domain-containing protein 1). In this context, our study provides important insights into the role of SCUBE-1, a novel ischemia marker, in late-onset FGR.

SCUBE-1 is a cell surface protein found on endothelial cells and platelets. It is released from platelets during hypoxia and under inflammatory conditions. It has been reported to increase in acute and chronic ischemic heart disease, and similarly, to show significant changes in clinical situations with an underlying ischemia, such as preeclampsia [10,11,12,13]. However, a PubMed search revealed no publications regarding its role in late-onset FGR. The basic hypothesis of our study was that maternal serum SCUBE-1 levels would be higher in late-onset FGR cases than in normal pregnancies. This study aimed to investigate the role of SCUBE-1, a new ischemia marker, in the diagnosis of late-onset FGR and its contribution to its pathophysiology.

## 2. Materials and Methods

For this prospective case–controlled study, women presenting to the obstetrics and gynecology and high-risk pregnancy outpatient clinic at our university hospital between July 2020 and July 2022 were evaluated for eligibility. Approval was obtained from the clinical research ethics committee of our university hospital. Thirty-three consecutive patients presenting to the outpatient clinic who met the criteria for late-onset FGR were included in the study group. After the study group was completed, 33 patients of a similar age and gestational age were selected from the normal pregnancy outpatient clinic for the control group. Written informed consent was obtained from all patients who agreed to participate in the study. The ethics committee meeting date was 29 June 2020 and the decision/acceptance number is 30.

The inclusion criteria for the study group were as follows: (1) consent to participate in the study, (2) age between 18 and 35 years old, (3) singleton pregnancy, (4) being at or after the 32nd week of gestation based on the last menstrual period with early-week sonographic measurements confirmed, and (5) an estimated birth weight measured ultrasonographically for late-onset FGR below the 10th percentile for gestational age. The control group was defined as having a similar gestational age to the study group but (1) consenting to participate, (2) aged between 18 and 35 years, (3) a singleton pregnancy, (4) being at or after the 32nd week of gestation based on the last menstrual period, with early-week sonographic measurements confirmed, and (5) an estimated birth weight measured ultrasonographically within the normal range for gestational age.

Cases were not accepted into the study if they had (1) pre-pregnancy hypertension (mild/moderate or overt blood pressure problems), (2) diabetes mellitus, (3) metabolic syndrome, (4) kidney disease, (5) liver disease, (6) heart disease, (7) endocrine disease, (8) malignant disease, (9) corticosteroid use, (10) multiple pregnancy, (11) fetal anomaly, (12) placental disease (cyst/tumor), (13) umbilical cord anomaly (single cord, etc.), (14) history of preterm premature rupture of membrane, (15) history of preeclampsia, (16) pregnancy achieved through assisted reproductive techniques, (17) did not want to participate into study, or (18) Current smoker. Only two cases initially agreed to participate in the study and passed the ultrasonographic evaluation stage, but were not included in the evaluation because they did not accept the blood sampling stage.

Fetal biometry (BPD (biparietal diameter), HC (Head circumference), FL (femur length), AC (abdominal circumference), EFW (estimated birth weight) measurements) and fetal/maternal vascular Doppler (fetal umbilical artery (FUA), fetal middle cerebral artery (FMCA), and maternal uterine artery (MUA)) parameters (Pulsatility index (PI)) were measured by one of the authors using an advanced ultrasonography device (Voluson E10 ultrasound system, GE, Tiefenbach, Austria) found in the inventory of the clinic where the study was conducted. Further details about the measurements were provided in our previous study [13]. Fetal sonography, Doppler study, and maternal blood sampling were performed on the same day. All sonographic measurements and maternal blood collection were performed by one of the authors (GD).

IMA was used instead of another known ischemia marker because our clinic has extensive research experience with IMA measurements in many other cases, and we have experience with the measurement technique [14,15,16,17,18,19,20,21,22]. Maternal serum SCUBE-1 and ischemia-modified albumin (IMA) levels were measured as described in our previous study [13]. Following an overnight fast, venous blood samples were collected from the forearm between 8:00 and 11:00 a.m. from pregnant women who met the sonographic evaluation and inclusion criteria. Following an overnight fast, a 5-mL serum sample was collected from the forearm into a gel-lined biochemistry tube with a serum separator. The blood samples were centrifuged at 3000 rpm for 10 min. Serum samples were then separated into Eppendorf tubes and stored at −80 °C for medical purposes. After all samples were collected, SCUBE-1 levels were measured using an ELISA kit in a medical biochemistry research laboratory. Maternal serum IMA measurement was performed using the colorimetric method based on albumin cobalt binding. Fetal cerebroplacental ratio was calculated by dividing the FMCA-PI value by the FUA-PI value.

Demographic data (age, gravida, parity, body mass index), pregnancy follow-up, and birth-related data (gestational week at inclusion in the study, gestational week at birth, delivery method, baby gender, birth weight, 5th minute Apgar score) for all cases were obtained from the hospital automated registration system.

For the study, data on age, gravida, parity, body mass index, gestational age at recruitment, gestational age at delivery, mode of delivery, neonatal gender, neonatal birth weight, 5-min APGAR, Neonatal intensive care unit requirement, Place of residence (Village or city), Socioeconomic status (Good, Moderate, Poor; based on women’s statement), Pregnancy complications that developed during follow-up (Gestational diabetes, Gestational hypertension, Pre-eclampsia, Preterm labor, Premature rapture of membrane), and Fetal complications (Respiratory distress syndrome, Jaundice, Anemia, Necrotizing enterocolitis, Newborn transient tachypnea) were obtained from the hospital’s automated registration system. In addition, ultrasonographic measurement data (Umbilical artery pulsatility index, mean cerebral artery pulsatility index, Cerebroplacental ratio, Uterine artery pulsatility index) and measured serum IMA and SCUBE-1 values were recorded for each patient and entered into SPSS 22 and analyzed. The Kolmogorov–Smirnov test was used to assess normality. Student’s *t*-test was used to compare the means and standard deviations of the normally distributed variables—serum ischemia-modified albumin, age, fetal middle cerebral artery pulsatility index, fetal cerebroplacental ratio, and fetal birthweight—between two groups. Since all other variables did not follow a normal distribution, the Mann–Whitney U test was used for comparison. The Fisher Exact Test is used to determine whether there is a statistically significant difference in the proportions of categories between two groups. The chi-square test was not used when the expected cell count was less than 5. *p* < 0.05 was considered statistically significant. Pearson correlation test was used for correlation analysis, and ROC analysis was used for sensitivity/specificity-cutoff value determination. There were no confounding factors or bias.

The G*Power 3.1.9.7 program was used for sample size and power analysis. Based on preliminary study results, when the effect size was set at 0.80 and 33 subjects were included in each group, the alpha value was 0.05 and the power was 0.94.

## 3. Results

A total of 66 third-trimester pregnant case data were analyzed within the scope of the study. There was no underlying cause in any of the FGR cases. The data of 33 cases with late-onset fetal growth restriction were compared with the data of 33 cases without any pregnancy complications, matched in terms of mean maternal age and gestational week at admission. The comparison of fetal and maternal clinical parameters of fetal growth restriction cases with the control group is shown in Table 2. Newborn birth weight and gestational age at delivery were found to be statistically significantly lower in the fetal growth restriction group compared with the control group. The fetal complications were more common in the FGR group than in the control group. The women in the FGR group were most commonly living in a village area.

The Comparison of maternal serum ischemia-modified albumin (IMA) level, maternal serum scube-1 level, fetal umbilical artery pulsatility index (FUA-PI), fetal middle cerebral artery pulsatility index (FMCA-PI), fetal cerebroplacental ratio (CPR), right and left maternal uterine artery pulsatility index (MUA-PI) data in cases with intrauterine fetal growth restriction compared to normal pregnancies is summarized in Table 2. In terms of comparing Doppler results, only the left maternal uterine artery Doppler PI and fetal umbilical artery PI value were found to be statistically significantly higher in FGR cases compared to control cases. In contrast, the calculated cerebroplacental ratio was considerably lower in FGR cases than in control cases. As shown in Table 3, when maternal serum was evaluated for oxidative stress parameters, although there was no statistically significant difference in IMA levels, substantial increases in SCUBE-1 levels, another new ischemia marker, were observed in the FGR group compared to the control group.

**Figure 1:**
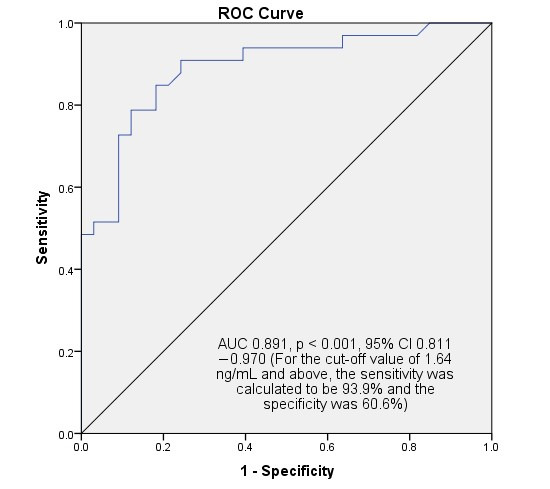
SPSS output for ROC analysis for predicting and diagnosing late-onset fetal growth restriction. In cases with a SCUBE-1 result of 1.64 ng/mL (cut-off value) and above, the sensitivity for fetal growth restriction was calculated to be 93.9% and the specificity was 60.6%.

In addition to the fact that maternal serum scube-1 value was found to be statistically significantly higher in late-onset FGR cases, FUA-PI (r = 0.396, *p* = 0.001, Pearson correlation test) and MUA-PI (r = 0.396, *p* = 0.001 for right, r = 0.306, *p* = 0.012 for left, Pearson correlation test) values showed positive correlation. In contrast, maternal serum scube-1 level showed negative correlations with CPR (r = −0.379, *p* = 0.002, Pearson correlation test) and newborn birth weight (r = −0.444, *p* = 0.001, Pearson correlation test). No other significant correlations were found in relation to serum SCUBE-1 and Doppler parameters. When serum SCUBE-1 measurement value was used as diagnostic cutoff for late-onset FGR, if a value of 1.64 ng/mL and above was detected, the sensitivity was calculated to be 93.9% and the specificity was 60.6% (AUC 0.891, *p* < 0.001, 95% CI 0.811–0.970, Figure 1). For this cut-off value, the calculated positive predictive value was 70.5% and the negative predictive value was 90.9%. A weak negative correlation, although not statistically significant, was found between oxidative stress parameters and gestational week (r = −0.224, *p* = 0.70 for SCUBE-1; r = −0.047, *p* = 0.708 for IMA). Also, a weak negative correlation was found between fetal birth weight and SCUBE-1 level (r = −0.444, *p* < 0.001).

## 4. Discussion

This clinical laboratory study aimed to contribute to understanding the pathogenesis of late-onset FGR, which accounts for a significant portion of intrauterine growth restriction and affects approximately 10% of pregnancies. The finding of significantly elevated serum SCUBE-1 levels in cases with FGR beyond 32 weeks suggests that oxidative stress parameters may be necessary for diagnosis, treatment, and delivery guidance in these cases. This new oxidative stress parameter showed high diagnostic sensitivity, especially for the diagnosis and follow-up of cases with fetal growth restriction, according to our study.

In humans, the SCUBE gene was first isolated from human endothelial cells. Three types have been identified. Scube-1, in particular, is thought to be stored in platelets and involved in endothelial-platelet adhesion. It is hypothesized to be released after induction and to contribute to platelet agglutination, thrombus formation, vascular occlusion, and oxidative stress [12]. Studies of SCUBE in obstetrics and gynecology are pretty limited. Previously, it had only been studied in cases of preeclampsia [13], gestational diabetes-related placental dysfunction [23], and ovarian torsion [24], and found to be statistically significantly higher in these conditions. In addition, it has been studied in many ischemic organ diseases and cancers [12]. Our study may be valuable because it presents the results of this new ischemia marker in cases of late-onset FGR. Thus, it may be incorporated into clinical practice and contribute to clinicians’ patient management processes.

During human pregnancy, dramatic cardiovascular and physiological changes occur to ensure fetal development. Consequently, uterine blood flow increases approximately 12-fold, while uterine resistance decreases, resulting in high-flow, low-resistance, and ongoing vasodilation in spiral arteries. Throughout this normal maternal adaptation process, endothelial-vascular smooth muscle coordination, extracellular matrix production/degradation, and communication with the trophoblast and immune system are fundamentally mediated by the nitric oxide signaling system and changes in angiogenic growth factors. The nitric oxide signaling system sustains this process by generating small to moderate amounts of reactive oxygen species (ROS). In cases of FGR, increased ROS and angiogenic mediators, resulting from increased NADPH oxidase activity, a component of the mitochondrial nitric oxide signaling system, cause hypertrophic effects on vascular walls. This process narrows the lumen of the mother’s vascular supply to the fetus, leading to endothelial dysfunction and chronic hypoxia, resulting in a small-for-gestational-age fetus. Several oxidative stress markers can be used to reveal this oxidative stress process [25,26]. However, none of them are mutually effective.

Platelet activation and aggregation are known to be important in the development of thrombosis. Concomitant endothelial dysfunction also contributes to this process, worsening it. Acute coronary artery disease is a prime example of this [27]. SCUBE-1 is known to be highly expressed in platelets, more so than in the endothelium, and to be stored in inactive platelets. Upon activation, it migrates to the platelet surface and contributes to thrombus formation. One study reported that SCUBE-1 functions as a platelet endothelial adhesion molecule and mediates the pathological process in cardiovascular diseases [28]. It is unclear whether increased SCUBE-1 levels are a cause or effect in cases of late-onset FGR. However, the lack of significant placental pathology in histological studies of placentas of cases of FGR that develop after 32 weeks of gestation supports the hypothesis that underlying vascular pathologies may be the primary trigger [29]. It may be speculated that late-onset FGR occurs when SCUBE-1-laden platelets adhere to endothelial surfaces damaged by processes such as infection or inflammation of unknown cause. In this way, placental oxidative stress can cause hypoperfusion and hypoxia, leading to late-onset FGR. In this case, increased SCUBE-1 may be more likely a consequence.

Mitochondrial dysfunction caused by oxidative stress has been reported to be associated with many pregnancy complications. Placental mitochondrial dysfunction may be significant in the development of adverse pregnancy outcomes such as intrauterine growth restriction, preeclampsia, preterm labor, and stillbirth [30]. Decreased mitochondrial DNA has been shown in cases of severe FGR [31]. Data from a systematic review [30] support the hypothesis that oxidative stress plays a role in the pathophysiology of fetal complications. Our previous study demonstrated that the level of SCUBE-1, a new marker of oxidative stress, was increased in preeclampsia [13]. In this study, we showed that SCUBE-1, but not IMA, is a novel oxidative stress marker that contributes to the pathophysiology of late-onset FGR.

We found elevated SCUBE-1 levels in late-onset FGR cases. This suggests that oxidative stress was increased in these cases, and the fetus remained small in response to oxidative stress. To support the pathophysiology, an experimental study administered glucocorticoids to pregnant women. This resulted in increased ROS levels, which in turn reduced the mitochondrial DNA content. All of this reduced mitochondrial ATP synthesis, reducing cellular energy [32]. Decreased cellular energy, coupled with the process of generating energy from food, may have contributed to the fetus remaining small. The initiating factor in late-onset FGR is unknown. Undiagnosed viral infection or subclinical inflammation may have initiated this process by causing placental endothelial damage, but further, larger series of studies are needed to support this pathophysiological hypothesis.

Contrary to popular belief, intrauterine growth restriction does not exert a protective effect on the brain in utero. In other words, in cases of early or late-onset symmetric/asymmetric FGR, brain morphological development is impaired, as in brain-bone development. Babies may be born with brain immaturity for gestational age. These babies have an increased risk of postnatal intraventricular hemorrhage, blood–brain barrier destabilization, cerebral white matter pathway abnormalities, and frontal lobe developmental delay [33]. All these data support the hypothesis that brain development is negatively affected in FGR cases. The current information indicates that the results of studies on the diagnosis, pathophysiology, follow-up, and screening of FGR cases would be significant. The presence of appropriate, effective biomarkers may reduce the extent of brain damage through early diagnosis and relevant, timely delivery planning. SCUBE-1, with its high diagnostic sensitivity, may be a helpful marker in this context.

Currently, numerous studies examine the relationship between placental ischemia and FGR [9]. In addition, no effective treatment options have been established for prophylaxis or treatment [34]. The only treatment options are prenatal lung-enhancing steroid injections and timing of delivery using sonography and Doppler parameter monitoring [35]. Oxidative stress parameters are not included in the management scheme in any of the current FGR follow-up treatment guidelines. The timing of delivery is the most crucial step in the treatment of late-onset FGR. No effective biochemical marker has been identified for this purpose. Our research suggested that the oxidative stress marker SCUBE-1, which is elevated in late-onset FGR cases, has high sensitivity for detecting FGR, may contribute to its pathophysiology, and may be helpful in this context in the future.

One study found that the level of IMA, a parameter of oxidative stress, increases throughout pregnancy, while malondialdehyde levels decrease [22]. Another study showed that IMA levels, which increase throughout pregnancy, were not associated with fetal weight. The study also demonstrated similar levels in the second and third trimesters [36]. Our study lacked data on changes in IMA and SCUBE-1 across pregnancy trimesters. This may be a limitation of our study. Furthermore, no significant change was found between gestational age and SCUBE-1 level in third-trimester cases. However, the negative correlation between SCUBE-1 levels and fetal birth weight may support the hypothesis that SCUBE-1 plays a role in the pathophysiology of late-onset fetal FGR.

Sterile inflammation in the tissue is generally considered beneficial. However, increased inflammation or a hyperimmune response disrupts the hemostatic balance, creating a chronic inflammatory process. Estrogen has been reported to be an immunomodulator and neuroprotective in ischemic stroke. However, the mechanism for this has not been clearly established [37]. Increased SCUBE-1 in FGR cases indicates the presence of an ischemic condition. Increased estrogen during pregnancy may have a protective effect on the placenta, potentially causing fetus to remain small to protect the fetus from more serious complications. Estrogen is known to significantly accelerate up to 35 weeks of gestation [38], and its protective effect may have been evident by 32 weeks, with late-onset FGR cases becoming evident at this week. Large-scale studies are needed to test this hypothesis.

The study did not compare cases of FGR at different gestational weeks. This raises questions about the generalizability of our study. Another limitation of our study is that it cannot specify at which gestational week the recommended SCUBE-1 level would yield better results. In addition to these limitations, our study suggests that this biochemical marker may be used to support the diagnosis in cases of late-onset FGR around 32 weeks of gestation, when fetal growth restriction is suspected. It is clear that further research is needed to recommend its wider and more effective use. From another perspective, SCUBE has three defined variants [12]. It is unclear which of these is the best marker. Our study provides results for only one of them. Further studies comparing all three markers could be planned.

Limitations of this prospective clinical study include the small number of FGR cases and the lack of genetic data. Another important limiting factor was the high sensitivity and low specificity. This low specificity may lead to some healthy cases being incorrectly diagnosed as FGR. This limitation could be addressed in future studies with a larger sample size. The lack of neonatal anthropometric data—length, head circumference, abdominal circumference, mid-arm circumference, clinical assessment of nutrition score, ponderal index, and relationship to gestational week—and the inability to correlate newborn babies with their intrauterine gestational week can be considered another limitation. The study was conducted at a single hospital, which limits its external validity. Future studies should include multi-center recruitment to account for regional or population-based variability in FGR and SCUBE-1 expression. Additionally, a comparison group of early-onset FGR could clarify whether SCUBE-1 elevation is specific to late-onset cases or a general feature of all FGR phenotypes; the lack of such comparison data also limits the generalizability of our study.

## 5. Conclusions

This study reported a significant increase in SCUBE-1 in late-onset FGR cases with a high level of sensitivity and low specificity. SCUBE-1 was an effective marker that could yield results from a maternal blood sample within an average of 1 h and may be helpful in the biochemical diagnosis of late-onset FGR.

## Figures and Tables

**Table 1:** Maternal factors and fetal outcomes in early-onset vs. late-onset fetal growth restriction, represented as odds ratios (Modified from reference number [7]).

	**Early-Onset vs. Late-Onset FGR**
Maternal factors	
Preeclampsia	OR 4.25, 95% CI 2.47–7.32
Gestational diabetes	OR 1.00, 95% CI 0.51–1.96
Cesarean delivery	OR 5.83, 95% CI 2.76–12.32
Vaginal Delivery	OR 0.63, 95% CI 0.13–2.95
Fetal factors	
APGAR score < 7 at five minutes	OR 6.35, 95% CI 2.98–13.56
Neonatal resuscitation	OR 6.11, 95% CI 3.08–12.12
NICU admission	OR 13.38, 95% CI 70–48.33
Anemia	OR 117.68, 95% CI 3.23–4289.12
Jaundice	OR 6.39, 95% CI 2.98–13.69
NEC	OR 12.77, 95% CI 3.00–54.40
Periventricular leukomalacia	OR 7.59, 95% CI 1.30–44.28
Intra-ventricular hemorrhage	OR 5.09, 95% CI 2.19–11.82
Respiratory distress syndrome	OR 7.08,95% CI 1.55–32.39
Sepsis	OR 10.92, 95% CI 2.57–46.34
Perinatal death	OR 10.01, 95% CI 5.76–17.39
Umbilical cord pH < 7.1	OR 1.57, 95% CI 0.94–2.61
Hypoglycemia	OR 2.12, 95% CI 0.91–4.94

OR: Odds ratio; NICU: neonatal intensive care unit; NEC: Necrotizing enterocolitis.

**Table 2:** Comparison of the cases with or without fetal growth restriction in terms of some maternal/fetal demographic factors.

KERRYPNX	Cases with FGR (n = 33)	Cases Without FGR (n = 33)	*p*
Age (years) ^a^	29.82 ± 5.66	30.15 ± 4.85	>0.05
Gravida (no.) ^b^	2.09 ± 1.23	2.55 ± 1.23	>0.05
Parity (no.) ^b^	0.91 ± 1.10	1.09 ± 1.40	>0.05
BMI (kg/m^2^) ^b^	27.04 ± 3.6	27.62 ± 2.9	>0.05
Gestational age at recruitment (weeks) ^b^	33.36 ± 2.97	33.39 ± 1.41	>0.05
Gestational age at delivery (weeks) ^b^	34.67 ± 3.67	38.48 ± 0.87	**<0.001**
Cesarean section (no.) ^c^	30 (90.9%)	24 (72.7%)	>0.05
Neonatal gender, male (no.) ^d^	14 (42.4%)	19 (57.6%)	>0.05
Neonatal birth weight (g) ^a^	2051.96 ± 466.89	3295.93 ± 345.80	**<0.001**
5-min APGAR score (no.) ^b^	7.32 ± 1.42	8.30 ± 1.24	>0.05
Place of residence (no.) ^d^			
Village	27 (81.8%)	14 (42.4%)	**0.001**
City	6 (18.2%)	19 (57.6%)	
Socioeconomic status (no.) ^c^			
Good	7 (21.2%)	14 (42.4%)	0.086
Moderate	13 (39.4%)	13 (39.4%)	
Poor	13 (39.4%)	6 (18.2%)	
Pregnancy complications that developed during follow-up (no.) ^c^			
Gestational diabetes	2 (6.1%)	2 (6.1%)	0.249
Gestational hypertension	2 (6.1%)	-	
Pre-eclampsia	1 (3.0%)	-	
Preterm labor	2 (6.1%)	-	
Premature rapture of membrane	1 (3.0%)	-	
Absent	25 (75.8%)	31 (93.9%)	
NICU requirement (no.) ^c^	22 (66.7%)	1 (3.0%)	**<0.001**
Fetal complications (no.) ^c^			
Respiratory distress syndrome	8 (24.2%)	-	**<0.001**
Jaundice	4 (12.1%)	2 (6.1%)	
Anemia	2 (6.1%)	-	
Necrotizing enterocolitis	1 (3.0%)	-	
Newborn transient tachypnea	7 (21.2%)	1 (3.0%)	
Absent	11 (33.3%)	30 (9.9%)	

FGR: Fetal growth restriction, BMI: Body Mass Index, no: Number, NICU: Neonatal intensive care unit. Mean ± standard deviation or number of cases (percentages in parentheses) values are given. ^a^ Student’s *t* test or ^b^ Mann–Whitney U test or ^c^ Fisher Exact Chi-square or ^d^ Chi-square test was used for comparison. The bold *p*-values refer to statistical significance.

**Table 3:** Comparison of the cases in terms of maternal serum ischemia markers (IMA and SCUBE-1) and some fetal/maternal Doppler ultrasound parameters.

	Patients with FGR (n = 33)	Control Group (n = 33)	*p*
Serum IMA (ABSU) ^a^	0.74 ± 0.64	0.73 ± 0.06	0.501
Serum SCUBE-1 (ng/mL) ^b^	3.45 ± 2.54	1.62 ± 0.50	**<0.001**
FUA-PI (no.) ^b^	1.29 ± 0.35	0.91 ± 0.11	**<0.001**
FMCA-PI (no.) ^a^	1.52 ± 0.32	1.61 ± 0.34	0.273
FCPR (no.) ^a^	1.45 ± 0.50	1.77 ± 0.37	**0.005**
Right MUA-PI (no.) ^b^	0.99 ± 0.41	0.69 ± 0.25	0.140
Left MUA-PI (no.) ^b^	1.00 ± 0.39	0.69 ± 0.21	**0.049**

FGR: Fetal growth restriction, ABSU: Absorbance unit, IMA: Ischemia-modified albumin, SCUBE-1: Signal peptide-CUB (complement C1r/C1s, Uegf, and Bmp1), FUA-PI: Umbilical artery Pulsatility index, FMCA-PI: Mean cerebral artery Pulsatility index, FCPR: Cerebro-placental ratio, MUA-PI: Uterine artery Pulsatility index, no: Number. ^a^ Student’s *t* test or ^b^ Mann–Whitney U test was used for comparison. The bold *p*-values refer to statistical significance. FUA-PI, FMCA-PI, FCPR, Right MUA-PI, Left MUA-PI are derived from calculated parameters.

## Data Availability

The original contributions presented in this study are included in the article. Further inquiries can be directed to the corresponding author.

## Abbreviations

The following abbreviations are used in this manuscript:

FGR	Intrauterine growth restriction
IMA	Serum ischemia-modified albumin
SCUBE-1	Signal peptide-CUB (complement C1r/C1s, Uegf, and Bmp1)-epidermal growth factor-domain-containing protein 1
FUA-PI	Fetal umbilical artery pulsatility index
FMCA-PI	Fetal middle cerebral artery pulsatility index
CPR	Fetal cerebroplacental ratio
MUA-PI	Maternal uterine artery pulsatility index
ROS	Reactive oxygen species
NICU	Neonatal intensive care unit
